# A urine-based ELISA with recombinant non-glycosylated SARS-CoV-2 spike protein for detecting anti-SARS-CoV-2 spike antibodies

**DOI:** 10.1038/s41598-023-31382-5

**Published:** 2023-03-16

**Authors:** Fernanda F. Ramos, Flávia F. Bagno, Paula F. Vassallo, João A. Oliveira-da-Silva, Thiago A. R. Reis, Raquel S. Bandeira, Amanda S. Machado, Daniela P. Lage, Vivian T. Martins, Ana P. Fernandes, Myron Christodoulides, Cecilia G. Ravetti, Vandack Nobre, Flávio G. da Fonseca, Eduardo A. F. Coelho, Fernanda Ludolf

**Affiliations:** 1 Programa de Pós-Graduação Em Ciências da Saúde: Infectologia E Medicina Tropical, Faculdade de Medicina, Universidade Federal de Minas Gerais, Belo Horizonte, MG 30.130-100 Brazil; 2 Centro de Tecnologia de Vacinas (CT Vacinas) / BH-Tec, Universidade Federal de Minas Gerais, Belo Horizonte, MG Brazil; 3 Hospital das Clínicas, Universidade Federal de Minas Gerais, Belo Horizonte, MG Brazil; 4 Departamento de Patologia Clínica, COLTEC, Universidade Federal de Minas Gerais, Belo Horizonte, MG Brazil; 5 Departamento de Análises Clínicas e Toxicológicas, Faculdade de Farmácia, Universidade Federal de Minas Gerais, Belo Horizonte, MG Brazil; 6 Neisseria Research Group, School of Clinical and Experimental Sciences, University of Southampton Faculty of Medicine, Southampton, England; 7 Departamento de Clínica Médica, Faculdade de Medicina, Universidade Federal de Minas Gerais, Belo Horizonte, MG Brazil; 8 Laboratório de Virologia Molecular e Aplicada, Departamento de Microbiologia, Instituto de Ciências Biológicas, Universidade Federal de Minas Gerais, Belo Horizonte, MG Brazil

**Keywords:** ELISA, Diagnostic markers, Viral infection, Applied immunology

## Abstract

Serological assays have been widely used to detect anti-SARS-CoV-2 antibodies, which are generated from previous exposure to the virus or after vaccination. The presence of anti-SARS-CoV-2 Nucleocapsid antibodies was recently reported in patients´ urine using an *in-house* urine-based ELISA-platform, allowing a non-invasive way to collect clinical samples and assess immune conversion. In the current study, we evaluated and validated another *in-house* urine-based ELISA for the detection of anti-SARS-CoV-2 Spike antibodies. Three partial recombinant SARS-CoV-2 Spike proteins comprising the Receptor Binding Domain, expressed in eukaryotic or prokaryotic systems, were tested in an ELISA platform against a panel of over 140 urine and paired serum samples collected from 106 patients confirmed positive for SARS-CoV-2 by qRT-PCR. The key findings from our study were that anti-SARS-CoV-2 Spike antibodies could be detected in urine samples and that the prokaryotic expression of the rSARS-CoV-2 Spike protein was not a barrier to obtain relatively high serology efficiency for the urine-based assay. Thus, use of a urine-based ELISA assay with partial rSARS-CoV-2 Spike proteins, expressed in a prokaryotic system, could be considered as a convenient tool for screening for the presence of anti-SARS-CoV-2 Spike antibodies, and overcome the difficulties arising from sample collection and the need for recombinant proteins produced with eukaryotic expression systems.

## Introduction

On March 11, 2020, the outbreak of coronavirus disease (COVID-19) caused by Severe Acute Respiratory Syndrome Corona Virus-2 (SARS-CoV-2) was classified as a pandemic^1^. In vitro diagnostics (IVDs) of assured quality, safety and performance were considered an essential component of a complete strategy to control the pandemic. IVDs for COVID-19 fall into two main categories: the direct detection of SARS-CoV-2 virus (RNA or protein) and indirect serological detection of anti-SARS-CoV-2 antibodies produced by the host's immune system^2–5^.

While serological tests should not be used for diagnosing an acute SARS-CoV-2 infection, they may indicate the presence of antibodies generated from a previous viral exposure or to vaccination. The presence of anti-SARS-CoV-2 antibodies has still not been recommended as a criterion to assess protection^6^. Today, serological results depend on the diversity of vaccines licensed in each country and the type of antigen used in the immunological tests, i.e. SARS-CoV-2 Nucleocapsid (N) and/or Spike (S) proteins, which may indicate antibodies from previous infection and/or vaccination^6,7^. As an antibody response to infection takes days to weeks to be detected reliably, serological assays have been more relevant for patients presenting for medical care with late-complications of illness and confirming persistent symptoms caused by ‘long-COVID’^8,9^.

Although considered minimally invasive and with a low rate of complication, venipuncture blood collection can be (i) unpleasant, especially for those who suffer with aichmophobia (fear of sharp objects), (ii) difficult to perform in some physical circumstances, and (iii) challenging in areas with limited and inaccessible healthcare resources^10,11^. The presence of anti-SARS-CoV-2 antibodies has been investigated in other biological fluids such as saliva and urine. Both biological fluids have advantages of non-invasive collection and self-collection at home^12–15^. For the purposes of detecting specific antibodies, urine collection is simpler and safer than serum preparation, and urine samples are easier to transport and are stable at 4 °C or room temperature for facile storage. Furthermore, saliva is a highly infectious fluid if collected in the active phase of the disease, and more caution is necessary for collection, handling and storage. Thus, the advantages of urine make it a convenient fluid for clinical and epidemiological studies, especially in places where access to patients is difficult^12,16^.

Urine-based tests to detect antibodies have been suggested as a non-invasive, simple and safe alternative to diagnose several infectious diseases, including COVID-19^12,16,17^. Recently, a urine based-ELISA assay able to detect anti-SARS-CoV-2 Nucleocapsid antibodies was developed and validated, with sensitivity of 94% and specificity of 100% after testing a collection of 209 samples from COVID-19 patients^12^. Among the four structural proteins of SARS-CoV-2, the N and S proteins are the most immunogenic and therefore the most used in serological assays^18^. The development of a urine-based SARS-CoV-2 S protein ELISA also may have an application in serological tests for the detection of vaccine-induced antibodies^12^.

The S protein is a large (~ 140 kDa) transmembrane surface glycoprotein containing two subunits, S1 and S2. The S protein is located on the surface of the virus and has been reported to be highly immunogenic. However, the high cost and limited production of full-length S protein poses considerable technical challenges. S1 contains mainly a Receptor Binding Domain (RBD), which mediates viral interaction with the angiotensin-converting enzyme 2 (ACE2) receptor on the host cell. Both the S1 subunit and its RBD domain are the main antigens used for S protein-based serological tests. It has also been reported that S1 exhibits less background and cross-reactivity compared with full-length S protein, which may be due to the lower similarity of the S1 subunit among the human coronaviruses compared to S2^19–22^.

Selecting suitable SARS-CoV-2 recombinant proteins is essential for developing a reliable serological test. SARS-CoV-2 N protein can be efficiently expressed with prokaryotic system(s) and it maintains good immunoreactivity. However, S protein has been expressed preferentially in eukaryotic systems, which often generate post-translational modifications of the antigen^23,24^. In terms of antigen-scale production, prokaryotic systems have advantages over eukaryotic systems, such as faster, more facile, higher productivity rates and significantly reduced costs. These advantages make such systems useful especially for the least-developed and low-to-middle income countries and in large-scale epidemiological investigations^23,25^.

In the current study, we tested the hypothesis that recombinant SARS-CoV-2 S proteins comprising the RBD expressed in eukaryotic or prokaryotic systems, could be used to detect anti-SARS-CoV-2 S antibodies in urine samples of individuals who had tested positive for SARS-CoV-2 by qRT-PCR assay.

## Material and methods

### Research subjects and biological samples

This study was approved by the Human Research Ethics Committee of the Federal University of Minas Gerais (UFMG, Belo Horizonte, Brazil) under protocol number CAAE 30,437,020.9.0000.5149 and complied with the provisions of the Declaration of Helsinki. All included participants were male or female adults who signed an informed consent form. The more complete description of patients is described elsewhere^12^.

Patients presenting with respiratory symptoms and seeking hospital assistance were assessed by the attending physician and included in this study after confirmation of SARS-CoV-2 infection with a positive qRT-PCR. Hospitalized patients (n = 104) were enrolled at the Hospital das Clínicas of UFMG and Hospital Santa Helena (Betim, Brazil). Non-hospitalized individuals (n = 2) who experienced mild COVID-19 symptoms and tested positive for SARS-CoV-2 by qRT-PCR were also included in this study and were enrolled via an active search in the general population (Belo Horizonte, Brazil). For this study, a total of n = 106 patients were enrolled.

Urine and paired serum samples from hospitalized patients were collected on the first day of inclusion and whenever possible, on days 1, 3, 7 and 14 after recruitment, thus varying the corresponding day Post Symptom Onset (PSO) for each patient. Urine and paired serum samples from non-hospitalized individuals were collected after ~ 20 days PSO. Samples collected before 2019 were considered truly negative and called “pre-COVID-19 negative”. Samples from individuals who had maintained a rigorous quarantine and did not show any symptoms, were considered theoretically negative and called “post-COVID-19 negative”. Both sample sets were used as negative controls. In total, urine samples used for the study were collected from 138 hospitalized and 2 non-hospitalized individuals (n = 140), and 19 pre- and 12 post-COVID-19 negative individuals (n = 31), respectively. In total, the serum samples were collected from 138 hospitalized and 2 non-hospitalized individuals (n = 140), and from 40 pre-COVID negative plus 6 post-COVID negative individuals (n = 46). All samples used in this study were collected before COVID-19 vaccination began in Brazil.

Urine and serum samples were collected as described by Ludolf et al*.,* (2022)^12^. Urine samples containing 0.1% (w/v) sodium azide were stored at 4ºC and serum samples were stored at − 20 °C until use.

### Production of recombinant SARS-CoV-2 S proteins

The amino acid sequences of the recombinant proteins expressed in the prokaryotic system, termed Prok1-S1 (250–667aa) and Prok2-S1 (319–591aa) and of the recombinant protein expressed in the eukaryotic system, termed Euk1-S1 (330–554aa), were compared by alignment to the whole SARS-CoV-2 S protein amino acid sequence (QIG55994.1) (Supplementary Fig. 1A). The Prok1-S1 sequence was based on the study of Woo et al*.*^26^ with respect to SARS-CoV-1, and Prok2-S1 was based on the study of Wrapp et al.^27^ for SARS-CoV-2. A partial rSARS-CoV-2 S protein antigen (330–554aa) (FPZ0537), which was expressed in mammalian Chinese Hamster Ovary (CHO) cells, was acquired from Fapon Biotech Inc., China.

The S1 subunit containing RBD domain regions of the SARS-CoV-2/human/BRA/SP02/2020 (MT126808.1) gene that encodes SARS-CoV-2 S protein (QIG55994.1) was partially cloned into a prokaryotic expression vector (Genescript®, USA). The pET-24a( +) vectors, containing the inserts corresponding to the amino acids (aa) sequences 250–667aa and 319–591aa were transformed into *Escherichia coli* BL21 strain cells (DE3), and recombinant protein expression was induced with Isopropyl β-D-1-ThioGalactopyranoside (IPTG; 0.5 mM) for 4 h in 2L of LB medium. The Prok1-S1 and Prok2-S1 proteins (with yields of 25 mg/L and 22 mg/L of culture, respectively) were purified on a HisTrap HP affinity column connected to an AKTA system (GE Healthcare, USA), following the manufacturer instructions. Inclusion bodies were solubilized in 8 M urea buffer. A 12.5% (w/v) SDS-PAGE was done to evaluate the purity of the recombinant protein (Supplementary Fig. 1B).

### Enzyme-linked immunosorbert assay (ELISA)

ELISA was done according to Ludolf et al. (2022)^12^, with a few modifications. ELISA 96-well high binding plates (Costar) were coated with 400 ng/well of rSARS-CoV-2 S proteins, Prok1-S1, Prok2-S1 or Euk1-S1, diluted in carbonate buffer, pH 9.6 for 18 h at 4 °C. The wells were blocked with a solution of Phosphate Buffered Saline containing 0.05% (v/v) Tween 20 (PBS-T) and 1% (w/v) bovine serum albumin (BSA) for 2 h at 25 °C. Then, 100 µL/well of urine (undiluted) or serum (1/100 dilution in PBS-T) samples were added to the wells and incubated for 1 h or 30 min, respectively, at 37 °C, after which they were again washed. Peroxidase-conjugated anti-human IgG antibody (Invitrogen A18811, 1/10,000 dilution in PBS-T), was added to the wells and the plates were incubated for 1 h or 30 min at 37 °C, for urine or serum samples, respectively. Next, the wells were washed and reactions were developed by addition of TMB (3,3',5,5-tetramethylbenzidine) for 20 min in the dark. Reactions were stopped by adding 0.5 M H_2_SO_4_ and the optical density (OD) values were read on a microplate spectrophotometer (Multiskan Go), at λ450 nm. The *cut-off* values were determined as the mean plus two times the standard deviation of negative samples for each assay. The index (I) value for each sample was calculated using the equation I = (ODλ450nm)/(cut-off). The index value was classified as positive above 1.1, indeterminate between 0.8 and 1.1 and negative below 0.8.

### Statistical analysis

Data were analyzed using the GraphPad PrismTM program (version 8.0 for Windows). Value distributions (mean (M) ± standard deviation (SD), as indicated) were obtained for continuous variables, while categorical ones were evaluated as proportions. Receiver-Operator Characteristic (ROC) curves were constructed with the OD values of the positive (SARS-CoV-2 infection) versus negative (pre-COVID-19 and post-COVID negative) samples. Diagnostic performance was evaluated by estimation of sensitivity (Se), specificity (Sp), Area Under the Curve (AUC) and Youden index (J). Confidence intervals (CI) were defined at the 95% confidence level (95% CI). A paired *t*-test was used to compare the distinct groups and *P* < 0.05 values were considered significant. Positive and Negative Predictive Values (PPV and NPV, respectively) were calculated based on the index value, excluding the indeterminate value samples, and using the equation NPV = true negative/false negative + true negative, and PPV = true positive/false positive + true positive.

## Results

We adapted an *in-house* urine-based ELISA protocol using rSARS-CoV-2 S proteins to evaluate the presence of anti–SARS-CoV-2 S antibodies in urine samples collected at different days Post Symptom Onset (PSO) from previously confirmed qRT-PCR positive patients. We used the well-established serum-based ELISA^28^ to compare efficiency. We evaluated the immunodiagnostic efficacy for COVID-19 of three rSARS-CoV-2 S proteins, one expressed in a eukaryotic system, Euk1-S1, and two expressed in a prokaryotic system, Prok1-S1 and Prok2-S1. For the analyses, urine and paired-serum samples from qRT-PCR–positive patients were used, as well as unpaired negative samples from pre–COVID-19 and post–COVID-19 individuals. All samples were collected before vaccination had started in Brazil.

### Evaluating anti-rSARS-CoV-2 Euk1-S1 antibodies in urine and paired serum samples

**Figure 1:**
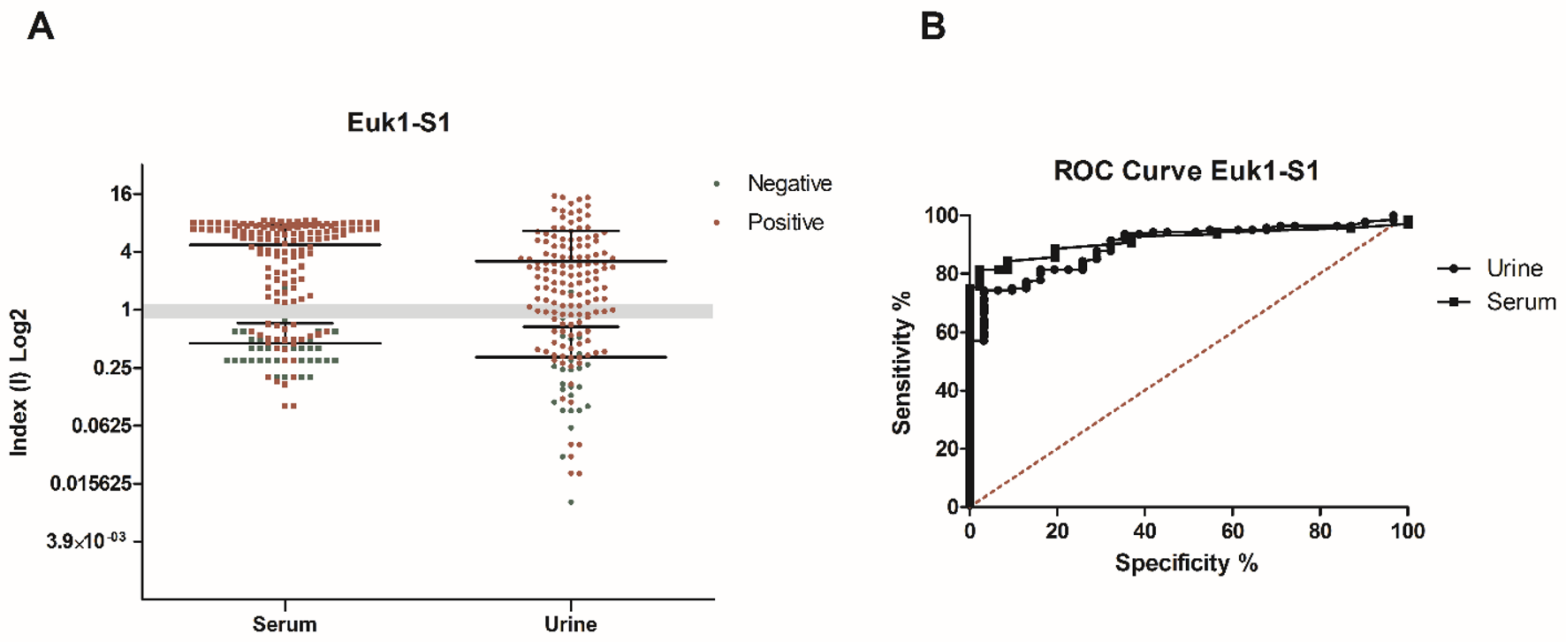
Comparative diagnostic performance of rSARS-CoV-2 Euk1-S1 protein with patient urine and serum samples. (**A**) ELISA assays were done using positive samples (n = 140 urine and n = 140 serum) from COVID-19 patients with previously positive qRT-PCR and negative samples (n = 31 urine and n = 46 serum). The mean of each group is shown and the gray band indicates indeterminate values for each sample, while index values below the range (< 0.8) are negative and values above (> 1.1) are considered positive. (**B**) Receiver Operating Characteristic (ROC) curves were constructed using the individual index (I) value for each sample, to obtain sensitivity, specificity and area under the curve values.

ELISA testing showed that 92/140 urine samples, collected from 106 qRT-PCR positive patients on different days PSO, reacted with the rSARS-CoV-2 Euk1-S1 protein with a positive index value of > 1.1, whereas 14 samples were classified as “indeterminate” (index values from 0.8 to 1.1) and 34 had a negative index value (< 0.8). Three indeterminate index value samples and one positive index value sample were observed from the 31 negative control urine samples (Fig. 1A). In parallel, 113/140 serum samples, collected from the 106 qRT-PCR positive patients on different days PSO, reacted with the rSARS-CoV-2 Euk1-S1 protein with a positive index value of > 1.1, whereas no sample was classified as “indeterminate” (index values from 0.8 to 1.1) and 27 samples had a negative index value (< 0.8). Only 2 indeterminate index value samples and 2 positive index value samples were observed from the 46 negative controls serum samples (Fig. 1A).

The individual index (I) values obtained by the OD (optical density)/cut-off ratios were used for standardization and comparison of the results. Sensitivity and specificity values of 67% and 97%, respectively, were calculated for urine samples tested in ELISA, and 81% and 98%, respectively, for serum samples. Comparative diagnostic performance of urine- and serum-based ELISA for COVID-19, under optimal experimental protocols for each biological specimen, is presented in Table 1. ROC curves were constructed and showed that the ELISAs had marginally inferior accuracy when urine was tested (AUC 0.8979) compared to serum (AUC 0.9151) (Fig. 1B and Table 1). PPV and NPV values were calculated based on the index value, which excluded the indeterminate index value samples, and were a NPV of 0.442 and PPV of 0.989 for urine and NPV of 0.675 and PPV of 0.939 for serum (Table 2).

**Table 1:** Comparative table of the results obtained from the diagnostic test for COVID-19 based on the search for specific IgG antibodies in urine and serum.

rSARS-CoV-2 antigen	Sample	n	AUC	*P* value	Cut-off	Se	95%CI	Sp	95%CI	J
Euk1-S1	URINE	+ 140/–31	0.8979	< 0.0001	> 1.035	67.14	58.70% to 74.84%	96,77	83.30% to 99.92%	0.64
	SERUM	+ 140/–46	0.9151	< 0.0001	> 1.150	81.43	73.98% to 87.50%	97.83	88.47% to 99.94%	0.79
Prok1-S1	URINE	+ 140/–31	0.9340	< 0.0001	> 1.015	80.71	73.19% to 86.89%	93.55	78.58% to 99.21%	0.74
	SERUM	+ 140/–46	0.8443	< 0.0001	> 1.020	62.86	54.29% to 70.87%	95.65	85.16% to 99.47%	0.58
Prok2-S1	URINE	+ 140/–31	0.9798	< 0.0001	> 1.065	89.29	82.94% to 93.88%	96.77	83.30% to 99.92%	0.86
	SERUM	+ 140/–46	0.7696	< 0.0001	> 1.010	40.00	31.82% to 48.61%	97.83	88.47% to 99.94%	0.38

Samples from symptomatic patients for Covid-19 and with PCR + for SARS-COV-2, as well as from healthy individuals pre-exposed to the virus were used. The individual index (I) value obtained by the Abs / *cut-off* ratio were used to construct ROC curves. The diagnostic performance of the antigen in relation to the type of sample used was based on the evaluation of sensitivity (95% CI), specificity (95% CI), area on the curve (AUC) and Youden index (J). Legend: J = (Se + Sp)–1; n = samples number, +  = positive sample, −  = negative sample.

**Table 2:** Predictive positive and predictive negative values of rSARS-CoV-2 S proteins for urine and serum samples.

rSARS-CoV-2 S antigen	Sample	PPV	NPV	Indeterminate
Euk1-S1	URINE	0.989	0.442	17
	SERUM	0.939	0.675	2
Prok1-S1	URINE	0.991	0.659	10
	SERUM	0.988	0.493	23
Prok2-S1	URINE	0.992	0.848	12
	SERUM	0.981	0.398	39

*PPV* and *NPV* were calculated based on the index value, excluding the indeterminate value samples and using the equation: *NPV* = true negative/false negative + true negative, and *PPV* = true positive/false positive + true positive.

### Anti-rSARS-CoV-2 Euk1-S1 stratified by PSO days

**Figure 2:**
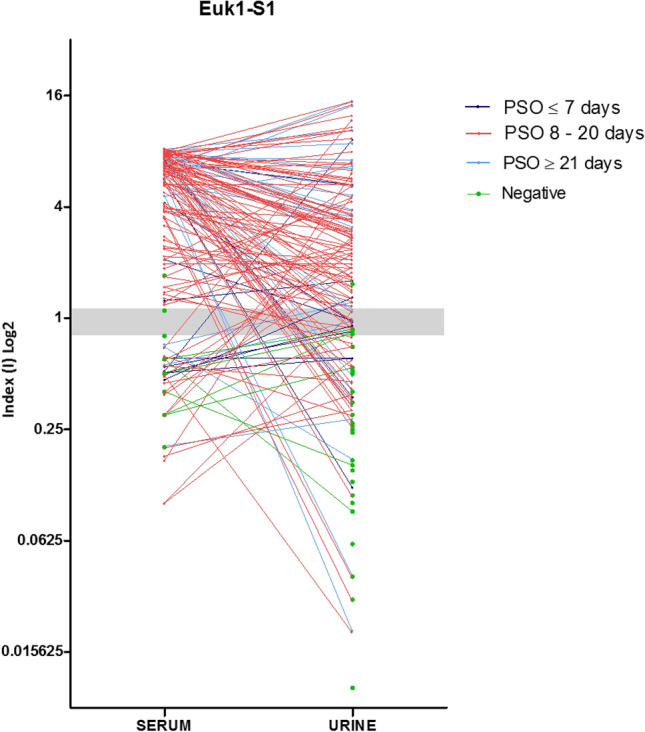
Evaluation of comparative urine and serum index values (I) for each patient according to the days Post-Symptoms Onset (PSO) by using rSARS-CoV-2 Euk1-S1 protein. The index values obtained from urine and serum samples for each patient are represented by circles and when paired they are interconnected by lines, each color being specific to the collection period after the onset of symptoms. Individual data were divided according to the PSO days of the collection date, i.e. at ≤ 7 days, 8 to 20 days and ≥ 21 days.

The comparison of reactive profiles using urine and serum samples against rSARS-CoV-2 Euk1-S1 protein was demonstrated in a stratified manner, according to PSO of ≤ 7 days, 8 to 20 days and ≥ 21 days (Fig. 2). All positive samples are paired and both samples collected on the same day. Up to 7 days PSO, 4/12 urine samples had a positive index value (> 1.1), while for serum, 6/12 samples had a positive index value. In the PSO period of 8 to 20 days, 72/103 urine samples and 86/103 serum samples had a positive index value. After 21 days PSO, 15/25 urine and 21/25 serum samples had a positive index value (Fig. 2).

### Evaluating anti-rSARS-CoV-2 Prok1-S1 antibodies in urine and paired serum samples

**Figure 3:**
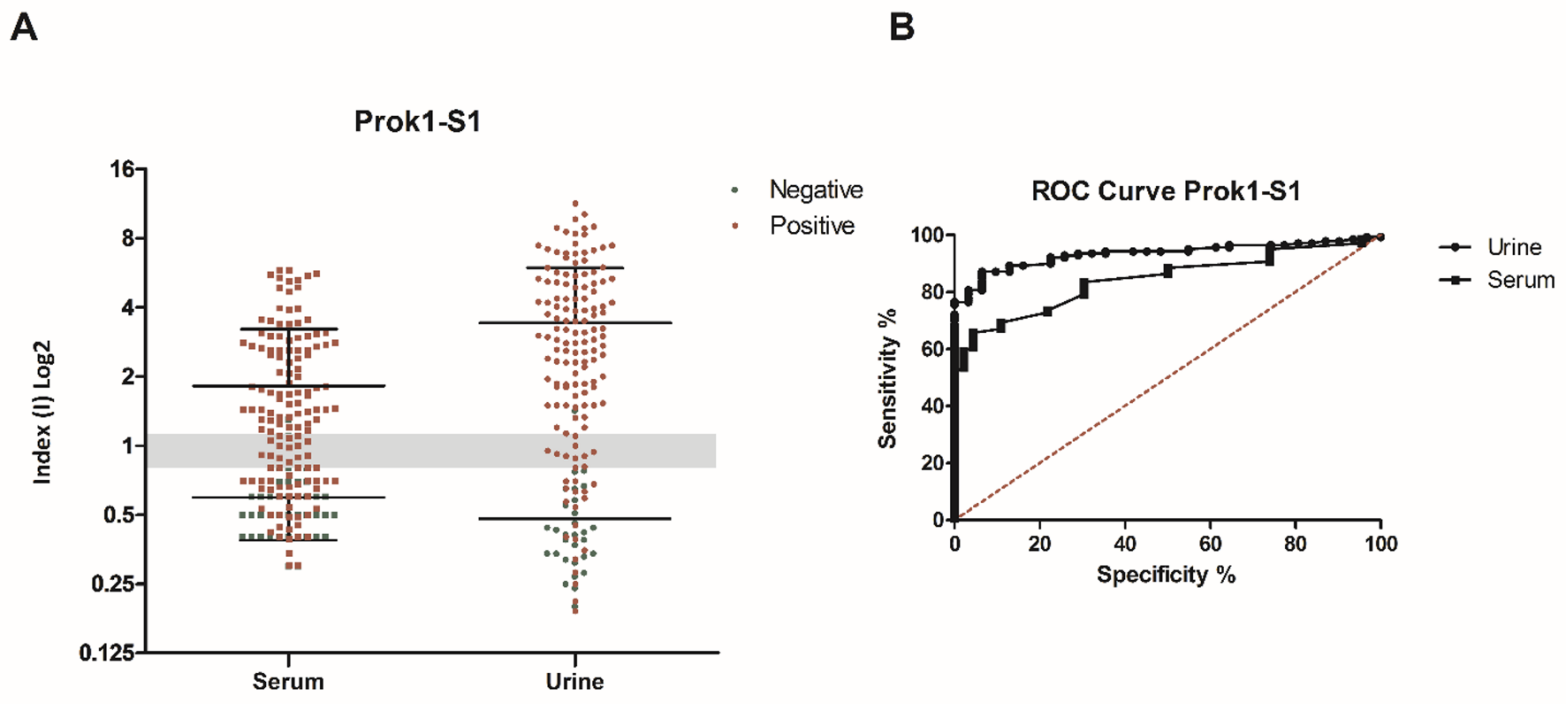
Comparative diagnostic performance of rSARS-CoV-2 Prok1-S1 protein with patient urine and serum samples. (**A**) ELISA assays were done using positive samples (n = 140 urine and n = 140 serum) from COVID-19 patients with previously positive qRT-PCR and negative samples (n = 31 urine and n = 46 serum). The mean of each group is shown and the gray band indicates indeterminate values for each sample, while index values below the range (< 0.8) are negative and values above (> 1.1) are considered positive. (**B**) Receiver Operating Characteristic (ROC) curves were constructed using the individual index (I) value for each sample, to obtain sensitivity, specificity and area under the curve values.

ELISA testing showed that 116/140 urine samples reacted with the rSARS-CoV-2 Prok1-S1 protein, showing a positive index value of > 1.1, whereas 9 samples were classified as “indeterminate” (index values from 0.8 to 1.1) and 15 had a negative index value (< 0.8). Only one indeterminate index value sample and one positive index value sample was observed within the group of 31 negative control urine samples (Fig. 3A). In parallel, 83/140 serum samples reacted with the rSARS-CoV-2 Prok1-S1 protein, showing a positive index value of > 1.1, whereas 17 samples were classified as “indeterminate” (index values from 0.8 to 1.1) and 40 had a negative index value (< 0.8). From the 46 negative control serum samples, 6 had indeterminate index values and 1 had a positive index value (Fig. 3A).

The individual index (I) values obtained by the OD (optical density)/cut-off ratio were used for standardization and comparison of the results. Sensitivity and specificity values of 80.71% and 93.55%, respectively, were calculated for urine samples tested in ELISA, and 62.86% and 95.65%, respectively, for serum samples. Again, comparative diagnostic performance of urine- and serum-based ELISA for COVID-19, under optimal experimental protocols for each biological specimen, is presented in Table 1. ROC curves were constructed and showed that the ELISA assays had superior accuracy when urine was tested (AUC 0.9340) compared to serum (AUC 0.8443) (Fig. 3B and Table 1). PPV and NPV values were calculated based on the index value, which excluded the indeterminate value samples, and were a NPV of 0.659 and a PPV of 0.991 for urine and a NPV of 0.493 and a PPV of 0.988 for serum (Table 2).

### Anti-rSARS-CoV-2 Prok1-S1 stratified by PSO days

**Figure 4:**
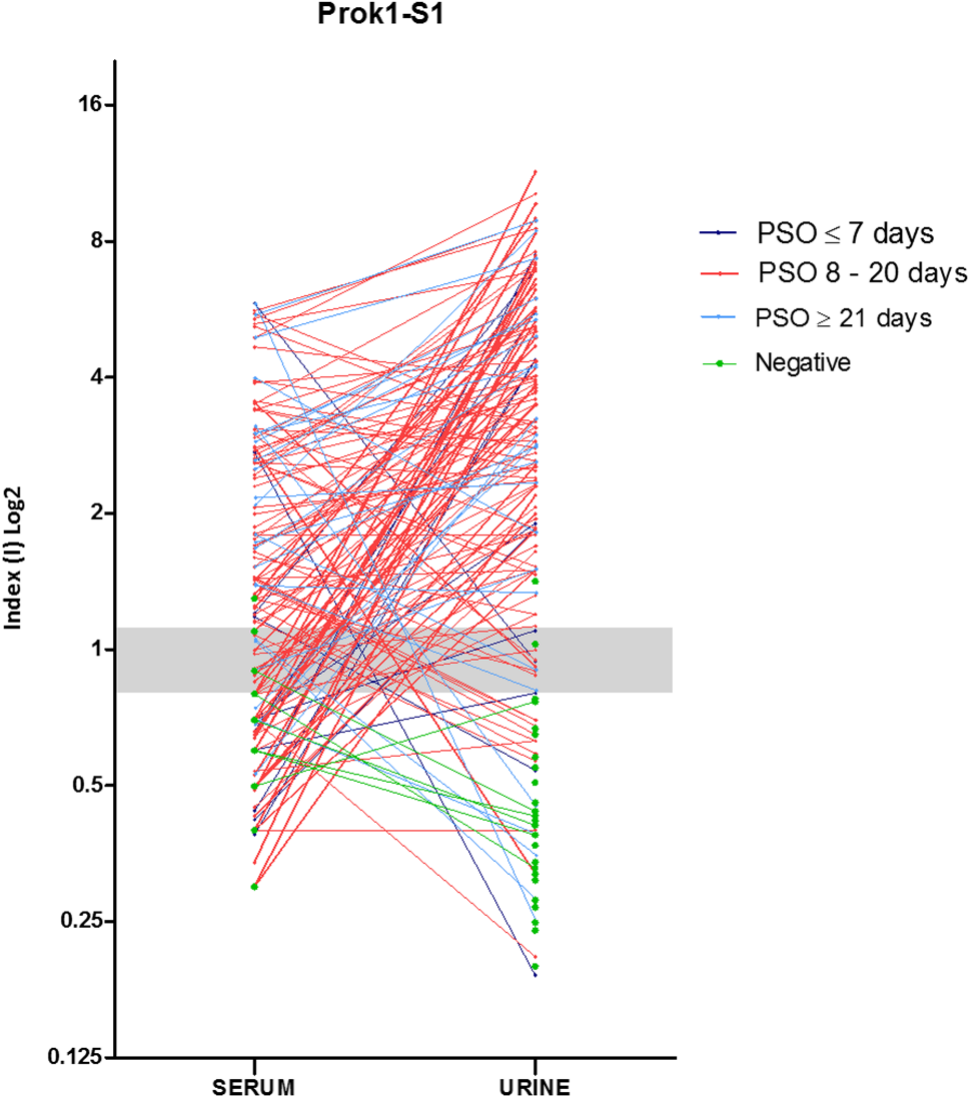
Evaluation of comparative urine and serum index values (I) for each patient according to the days post-symptoms onset (PSO) by using rSARS-CoV-2 Prok1-S1 protein. The index values obtained from urine and serum samples for each patient are represented by circles and when paired they are interconnected by lines, each color being specific to the collection period after the onset of symptoms Individual data were divided according to the PSO days of the collection date, i.e. at ≤ 7 days, 8 to 20 days and ≥ 21 days.

Comparison of reactive profiles of urine and serum samples against rSARS-CoV-2 Prok1-S1 protein was demonstrated in a stratified manner according to PSO of ≤ 7 days, 8 to 20 days and ≥ 21 days (Fig. 4). All positive samples are paired and collected on the same day. Up to 7 days PSO, 8/12 urine samples and 4/12 serum samples had a positive index value (> 1.1). In the PSO period of 8 to 20 days, 87/103 urine samples and 61/103 serum samples had a positive index value. After 21 days PSO, 21/25 urine and 18/25 serum samples had a positive index value (Fig. 4).

### Evaluating rSARS-CoV-2 Prok2-S1 antibodies in urine and paired serum samples

**Figure 5:**
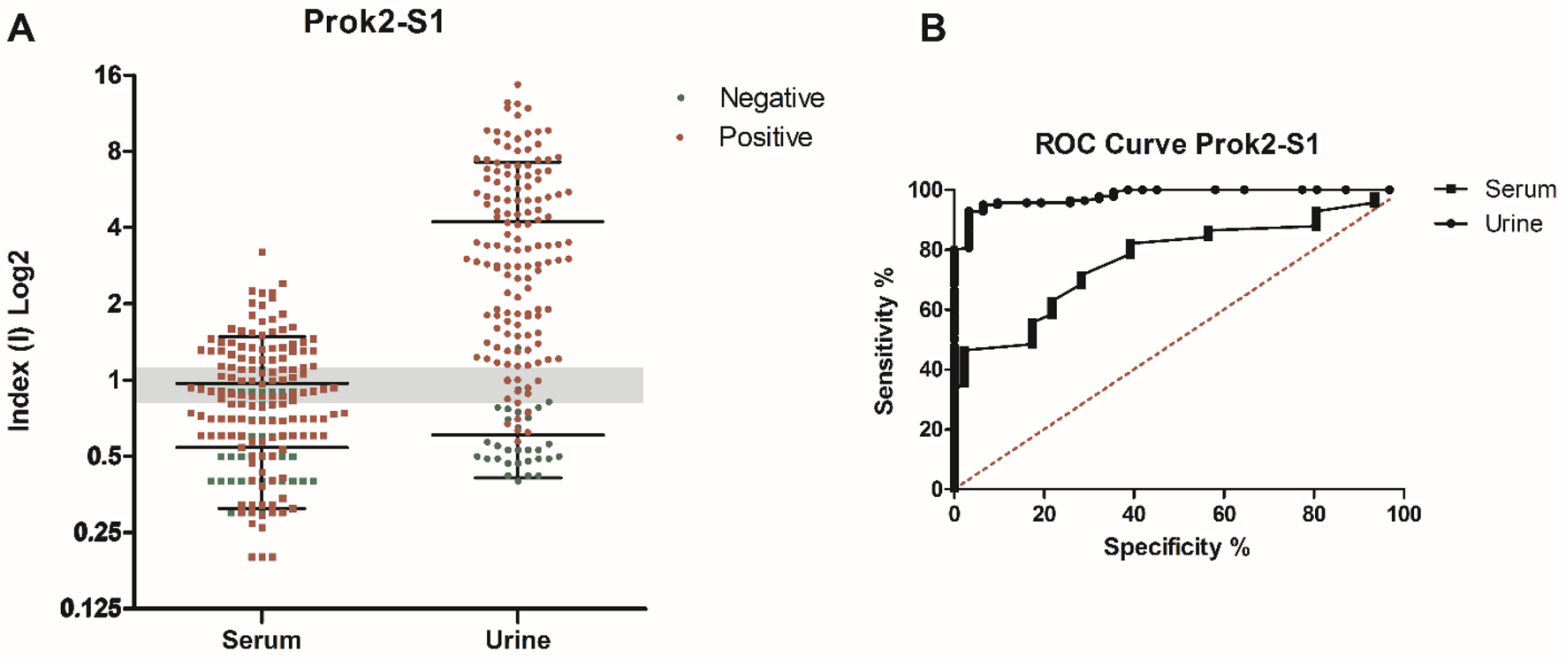
Comparative diagnostic performance of rSARS-CoV-2 Prok2-S1 protein with patient urine and serum samples. (**A**) ELISA assays were done using positive samples (n = 140 urine and n = 140 serum) from COVID-19 patients with previously positive qRT-PCR and negative samples (n = 31 urine and n = 46 serum). The mean of each group is shown and the gray band indicates indeterminate values for each sample, while index values below the range (< 0.8) are negative and values above (> 1.1) are considered positive. (**B**) Receiver Operating Characteristic (ROC) curves were constructed using the individual index (I) value for each sample, to obtain sensitivity, specificity and area under the curve values.

ELISA testing showed that 125/140 urine samples reacted with the rSARS-CoV-2 Prok2-S1 protein, showing a positive index value of > 1.1, whereas 10 samples were classified as “indeterminate” (index values from 0.8 to 1.1) and 5 had a negative index value (< 0.8). From the 31 negative controls urine samples, 2 had an indeterminate index value and there was only one sample with a positive index value (Fig. 5A). In parallel, 53/140 serum samples reacted with the rSARS-CoV-2 Prok2-S1 protein, showing a positive index value of > 1.1, whereas 31 samples were classified as “indeterminate” (index values from 0.8 to 1.1) and 56 had a negative index value (< 0.8). From the 46 negative controls serum, 8 samples had an in indeterminate index value and one had a positive index value (Fig. 5A).

The individual index (I) value obtained by the OD (optical density) / cut-off ratio were used for standardization and comparison of the results. Sensitivity and specificity values of 89.29% and 96.77%, respectively, were calculated for urine samples tested in ELISA, as well as 40.00% and 97.83 respectively, for serum samples. Again, comparative diagnostic performance of urine- and serum-based ELISA for COVID-19, under optimal experimental protocols for each biological specimen, is presented in Table 1. ROC curves were constructed and showed that the ELISA assays showed superior accuracy when urine was tested (AUC 0.9798) compared to serum (AUC 0.7696) (Fig. 5B and Table 1). PPV and NPV values were calculated based on the index value, which excluded the indeterminate value samples, and were a NPV of 0.848 and a PPV of 0.992 for urine and a NPV of 0.398 and a PPV of 0.981 for serum (Table 2).

### Anti-rSARS-CoV-2 Prok2-S1 stratified by PSO days

**Figure 6:**
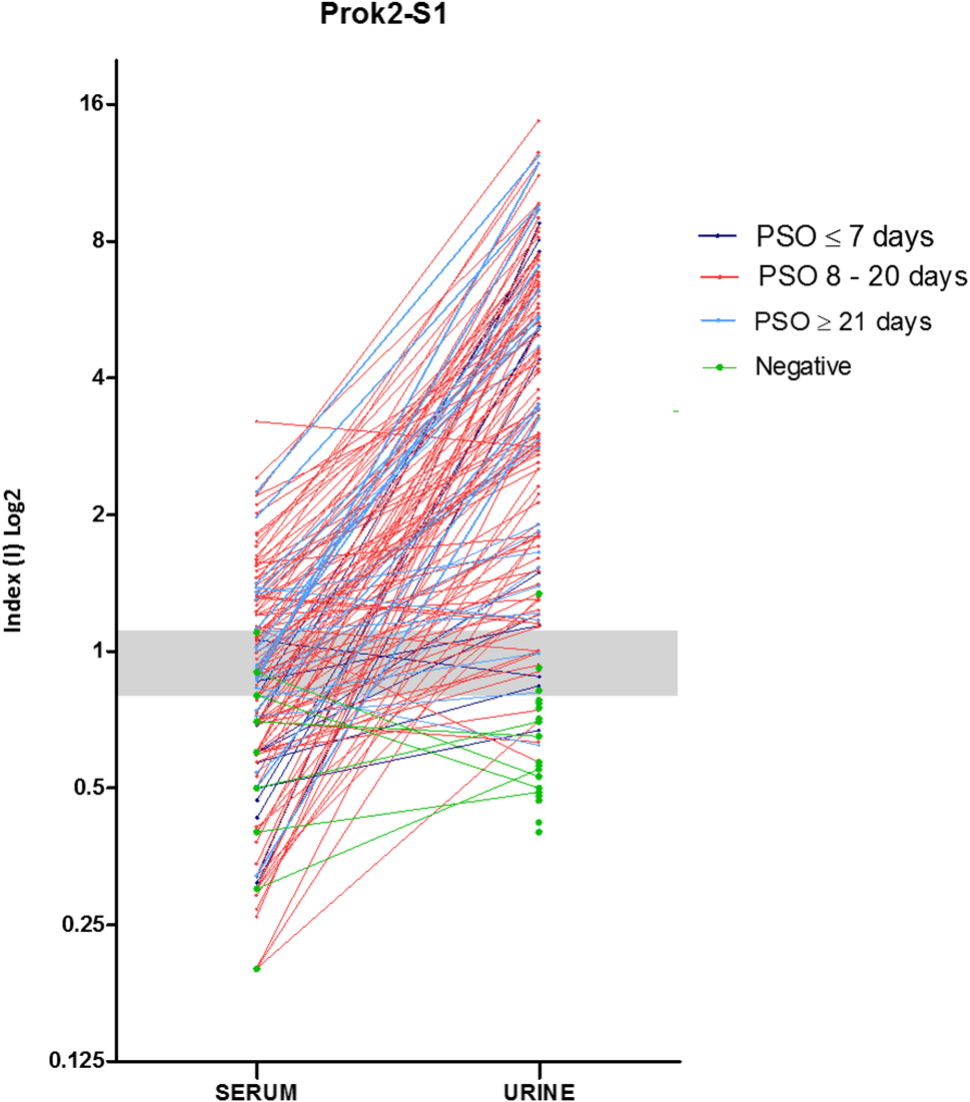
Evaluation of comparative urine and serum index values (I) of each patient according to the days post-symptoms onset (PSO) by using rSARS-CoV-2 Prok2-S1 protein. The index values obtained from urine and serum samples for each patient are represented by circles and when paired they are interconnected by lines, each color being specific to the collection period after the onset of symptoms Individual data were divided according to the PSO days of the collection date, i.e. at ≤ 7 days, 8 to 20 days and ≥ 21 days.

Comparison of reactive profiles of urine and serum samples against rSARS-CoV-2 Prok2-S1 protein was demonstrated in a stratified manner according to PSO of ≤ 7 days, 8 to 20 days and ≥ 21 days (Fig. 6). All positive samples are paired and collected on the same day. Up to 7 days PSO, 9/12 urine samples and 0/12 blood samples had a positive index value (> 1.1). In the PSO period of 8 to 20 days, 94/103 urine samples and 45/103 serum samples had a positive index value. After 21 days PSO, 22/25 urine and 08/25 serum samples had a positive index value (Fig. 6).

## Discussion

The main findings from the current study are that rSARS-CoV-2 S proteins could discriminate serologically positive COVID-19 patients using an *in-house* urine-based ELISA platform. To our knowledge, our study is the first to show the presence of these antibodies in urine samples, collected on days between 2 and 38 PSO from COVID-19 qRT-PCR positive patients, using recombinant S proteins expressed in either a eukaryotic or prokaryotic system. It is noteworthy that the comparative use of paired serum samples as reference was fundamental for this study, as serum-based ELISA is a well-established platform.

We calculated sensitivity (67.14% and 81.43%) and specificity (96,77% and 97.83%) values for the urine and paired-serum, respectively, when eukaryotic-expressed rSARS-CoV-2 Euk1-S1 protein, was used. However, we observed significantly lower sensitivity in serum-based ELISA assays for prokaryote-expressed SARS-CoV-2 S protein (62.86% Prok1-S1 and 40.00% Prok2-S1, respectively), compared to the eukaryotic-expressed SARS-CoV-2 Euk1-S1 protein (81.43%). Thus, prokaryote-expressed SARS-CoV-2 S protein may be not suitable for detecting anti-SARS-CoV-2 antibodies in serum. However, we found significantly higher sensitivity when using the prokaryote-expressed SARS-CoV-2 proteins Prok1-S1 (80.71%) and Prok2-S1 (89.29%) in the urine-based ELISA platform. These findings suggest that a urine-based ELISA with prokaryote-expressed rSARS-CoV-2 proteins (Prok1-S1 and Prok2-S1) could be a convenient tool for screening large numbers of people for the presence of anti-SARS-CoV-2 S antibodies and overcoming the difficulties and costs arising from sample collection and the production of recombinant protein(s). We did not find any studies or commercial serological tests using the S protein expressed in a prokaryotic system, and protein expression in a eukaryotic system appears to be the norm. Our study suggests that using eukaryotic recombinant SARS-CoV-2 S protein was not a critical requirement to obtain relatively high serology efficiency in our *in-house* urine-based assay.

Overall, IgG SARS-CoV-2 antibodies appear to peak at 14–30 days PSO and then slowly decline for 2–3 months^29,30^. The degree and duration of immunity that antibodies may confer, from infection or vaccination, remain unclear. Commercial serum-based antibody tests show low accuracy in the early stages of infection, since the immune response is still developing^5,11,31,32^. This rise in IgG production during the first days PSO was demonstrated recently with an *in-house* SARS-CoV-2 Nucleocapsid (N) urine-based ELISA^12^. Our findings are consistent for the SARS-CoV-2 S protein urine-based ELISA, where most of the false negatives were found in the collection range before the 7^th^ day PSO. Immune conversion of IgG antibodies in urine and serum samples, with an increase in IgG levels along the PSO days, is represented by two patients in Supplementary Fig. 2. Although we have worked to optimize our *in-house* ELISA tests, we were unable to achieve sensitivity performance figures like tests reported in the literature or commercially available^2^. This is probably because we have included samples with early PSO days collections, i.e. before immune conversion. As speculated previously, the relatively late IgG response to the S protein, compared to the IgG response to the N protein, in with SARS-CoV-2 infected individuals, might explain the differences in sensitivity achieved^23^. This late IgG response might also explain the negative index values obtained for some of the patients with both urine and serum samples collected on days 26, 27 and 28 PSO, and for both prokaryotic recombinant proteins, Prok1S1 and Prok2-S1. Although we identified antibodies in patients up to 38 days PSO, a longer follow-up study is still necessary to establish the diagnostic detection window of anti–rSARS-CoV S antibodies in urine samples. Experimental replication of urine and serum samples from same patient were done to assess reproducibility and the results showed similar indices amongst the replicates (Supplementary Fig. 3).

The specificity of an immunodiagnostic test for COVID-19 may be estimated by testing samples collected before the emergence of SARS-CoV-2^2^ and before mass vaccination in the case of SARS-CoV-2 S protein. Urine collection has been neglected as a biological specimen as urine-based immune tests are not usually available. Our study included 19 urine samples collected in 2019 before the outbreak and 12 urine samples collected during 2021 from individuals who maintained a rigorous quarantine regimen and who did not show any symptoms throughout 2020/2021. The collection of the samples was done during ‘lockdown’ with distancing protocols recommended, which made it logistically more difficult to collect samples from non-hospitalized patients. We were only able to include paired urine and serum sample from two non-hospitalized patients. However, it is important to state that we had not made any comparison between these two groups and included both as RT-qPCR positive patients. Another limitation of our study was that the urine-based ELISA was not tested with samples obtained from patients with other diseases, including infection with other coronaviruses and, therefore, we cannot rule out the possibility of cross-reactivity. Nevertheless, we suggest that the SARS-CoV-2 S protein was specific for COVID-19 in our urine-based ELISA, since we observed only one false positive for all three proteins from 31 negative individuals using the rSARS-CoV-2 Euk1-S1, Prok1-S1 and Prok2-S1 proteins.

Patients included in our study were unvaccinated at the time of sample collection, and we can infer that the antibodies detected with our urine-based ELISA most likely were generated from exposure to the virus. Future studies could seek to identify vaccine-induced anti-SARS-CoV-2 S antibodies in urine, particularly because most approved vaccines use SARS-CoV-2 S protein to elicit protective immunity. Anti-SARS-CoV-2 S antibodies elicited after COVID-19 and/or immunization with an inactivated vaccine have been identified by serum-based ELISA, using the same rSARS-CoV-2 Euk1-S1 protein as used in our study^28^, indicating the potential of identifying vaccine-generated antibodies by our urine-based ELISA. It is worth noting that the antibodies were identified in samples collected in 2020 and as the S protein continues to mutate, future studies are probably still needed to establish assay sensitivity in patients infected with SARS-CoV-2 variants. As our understanding of immunity and correlates of protection increases, and the variety of immunoassays multiplies, different assays will be used to answer particular questions^33^. The presence in urine of anti-SARS-CoV-2 antibodies to both S and N proteins^12^ suggests the development of a more complete platform, with each antigen (separately or together) used to address specific questions.

Antigen selection, source, and purity are crucial parameters for immunoassay development as they determine efficiency, fields of application and mass production. Prokaryotic or eukaryotic expression systems are used to produce recombinant proteins. The formers have significant production advantages, but are potentially limited by the lack of post-translational modification^34^. SARS-CoV-2 S protein is extensively glycosylated with at least 24 host-derived N- or O- glycan sites. The virus acquires glycan by appropriating the glycosylation machinery in the host cell reticulum-golgi intermediate compartments when offspring virions are formed^35^. To our knowledge, there is no available serological test or published study on detecting anti-SARS-CoV-2 S antibodies using prokaryotic-expressed rSARS-CoV-2 protein, which is probably due to the influence of post-translational modification for diagnostic performance. Thus, rSARS-CoV-2 S protein as a transmembrane surface glycoprotein has been expressed using a eukaryotic expression system, which often retains glycan post-translational modifications of the antigen, unlike bacterial recombinant proteins- or peptide- based ELISAs^23,24^. We found a higher sensitivity for prokaryotic-expressed rSARS-CoV-2 S proteins over eukaryotic-expressed rSARS-CoV-2 S proteins when using a urine-based ELISA. Although we have proven the applicability of the use of non-glycosylated SARS-CoV-2 S proteins in ELISA assays using urine samples, we could not find any explanation in the literature for why this recognition of non-glycosylated proteins occurs when using urine instead of serum. Urea has been used in ELISA for dissociation of low affinity antibody, allowing only high affinity antibodies to maintain reactivity. We hypothesize that the presence of urea in urine samples could be influencing the test, as the normal range of urea in human blood is 2–6 mM and 50 times more concentrated in urine^36–38^. To test this hypothesis, we did an assay using the Prok2-S1 proteins with serum samples diluted (1/100) in PBS-T, PBS-T with 50 mM Urea, PBS-T with 100 mM urea and in urine collected before the pandemic. We found no differences in recognition amongst the different sample diluents used, including those samples from RT-qPCR positive patients who had positive reactivity when using urine and negative when using serum (Supplementary Fig. 4). Thus, our data suggest that non-glycosylated SARS-CoV-2 S proteins could be used to assay seroconversion, overcoming many of the problems associated with eukaryotic expression of protein. Until now, protein S glycosylation appeared to be essential for serological testing, and since the beginning of the pandemic there have been significant studies reporting the development of SARS-CoV-2 S protein constructs that allow for greater recombinant protein production and protein stability. Different expression systems impact on glycosylation of the SARS-CoV-2 S glycoproteins by the type of attached glycan^39^, the production yields and even batch-to-batch variation^34,40^. Recombinant proteins, particularly S1, are one of the key reagents used to produce immunoassays for detecting IgG, IgM or IgA antibodies to SARS-CoV-2. However, expressing S1 protein in the correct conformation is challenging, and in some cases, the antibodies that recognize the S protein are unable to bind recombinant S protein^41^. In addition, high cost and insufficient capacity to produce enough glycosylated recombinant S1 proteins may limit accessibility to the immunoassay in low or middle income regions of world^42^.

The findings from our study could be applied to other diseases that are diagnosed serologically using pathogen glycosylated antigens. This is especially true for neglected tropical diseases: for example, disrupting the carbohydrate molecules of the parasitic roundworm *Strongyloides stercoralis* caused a significant reduction in immunoassay detection. This is consistent with the hypothesis that specific immune reactivity is reduced when glycosylated epitopes are removed from parasite antigens, as demonstrated in immunoassays with sera from patients with *Hemonchus contortus, Taenia solium, Leishmania spp.* and *S. stercoralis*^43^.

## Conclusions

Among the many different diagnostic tools available^44^, it is very important to be aware of the objective for which each diagnostic platform has been developed, to understand their performance, characteristics, and limitations. There are some future steps that must be taken to transform the present *in house* urine-based ELISA into a community tool. Despite the many advantages of ELISA^12^, it still a laboratory-based technique and other platforms may be considered. For example, there are many Point-Of-Care rapid sensor tests in use for COVID-19 detection based on sampling a drop of whole blood. Demonstration of the accuracy of urine as a sample in these rapid tests could offer an unintrusive means to detect COVID-19.

In summary, our proof-of-concept study suggests that a urine-based ELISA using prokaryotic-expressed S proteins could provide a convenient strategy for screening large numbers of people for the presence of S protein antibodies, and overcome the difficulties and costs arising from serum sampling and the production of recombinant proteins with eukaryotic expression systems.

## Supplementary Information

Supplementary Information.

## Data Availability

The authors report that all data supporting the findings of this study are presented within the paper.
