# Supplementary material for: A urine-based ELISA with recombinant non-glycosylated SARS-CoV-2 spike protein for detecting anti-SARS-CoV-2 spike antibodies
Source: Sci Rep. 2023 Mar 16;13:4345. doi: 10.1038/s41598-023-31382-5 (PMC10018619; doi:10.1038/s41598-023-31382-5)
Supplement: Supplementary file 1 — Supplementary Information. [file 41598_2023_31382_MOESM1_ESM.docx]

A

MFVFLVLLPLVSSQCVNLTTRTQLPPAYTNSFTRGVYYPDKVFRSSVLHSTQDLFLPFFSNVTWFHAIHVSGTNGTKRFDNPVLPFNDGVYFASTEKSNIIRGWIFGTTLDSKTQSLLIVNNATNVVIKVCEFQFCNDPFLGVYYHKNNKSWMESEFRVYSSANNCTFEYVSQPFLMDLEGKQGNFKNLREFVFKNIDGYFKIYSKHTPINLVRDLPQGFSALEPLVDLPIGINITRFQTLLALHRSYLTPGDSSSGWTAGAAAYYVGYLQPRTFLLKYNENGTITDAVDCALDPLSETKCTLKSFTVEKGIYQTSNFRVQPTESIVRF**PNITNLCPFGEVFNATRFASVYAWNRKRISNCVADYSVLYNSASFSTFKCYGVSPTKLNDLCFTNVYADSFVIRGDEVRQIAPGQTGKIADYNYKLPDDFTGCVIAWNSNNLDSKVGGNYNYLYRLFRKSNLKPFERDISTEIYQAGSTPCNGVEGFNCYFPLQSYGFQPTNGVGYQPYRVVVLSFELLHAPATVCGPKKSTNLVKNKCVNFNFNGLTGTGVLTE**SNKKFLPFQQFGRDIADTTDAVRDPQTLEILDITPCSFGGVSVITPGTNTSNQVAVLYQDVNCTEVPVAIHADQLTPTWRVYSTGSNVFQTRAGCLIGAEHVNNSYECDIPIGAGICASYQTQTNSPRRARSVASQSIIAYTMSLGAENSVAYSNNSIAIPTNFTISVTTEILPVSMTKTSVDCTMYICGDSTECSNLLLQYGSFCTQLNRALTGIAVEQDKNTQEVFAQVKQIYKTPPIKDFGGFNFSQILPDPSKPSKRSFIEDLLFNKVTLADAGFIKQYGDCLGDIAARDLICAQKFNGLTVLPPLLTDEMIAQYTSALLAGTITSGWTFGAGAALQIPFAMQMAYRFNGIGVTQNVLYENQKLIANQFNSAIGKIQDSLSSTASALGKLQDVVNQNAQALNTLVKQLSSNFGAISSVLNDILSRLDKVEAEVQIDRLITGRLQSLQTYVTQQLIRAAEIRASANLAATKMSECVLGQSKRVDFCGKGYHLMSFPQSAPHGVVFLHVTYVPAQEKNFTTAPAICHDGKAHFPREGVFVSNGTHWFVTQRNFYEPQIITTDNTFVSGNCDVVIGIVNNTVYDPLQPELDSFKEELDKYFKNHTSPDVDLGDISGINASVVNIQKEIDRLNEVAKNLNESLIDLQELGKYEQYIKWPWYIWLGFIAGLIAIVMVTIMLCCMTSCCSCLKGCCSCGSCCKFDEDDSEPVLKGVKLHYT

B


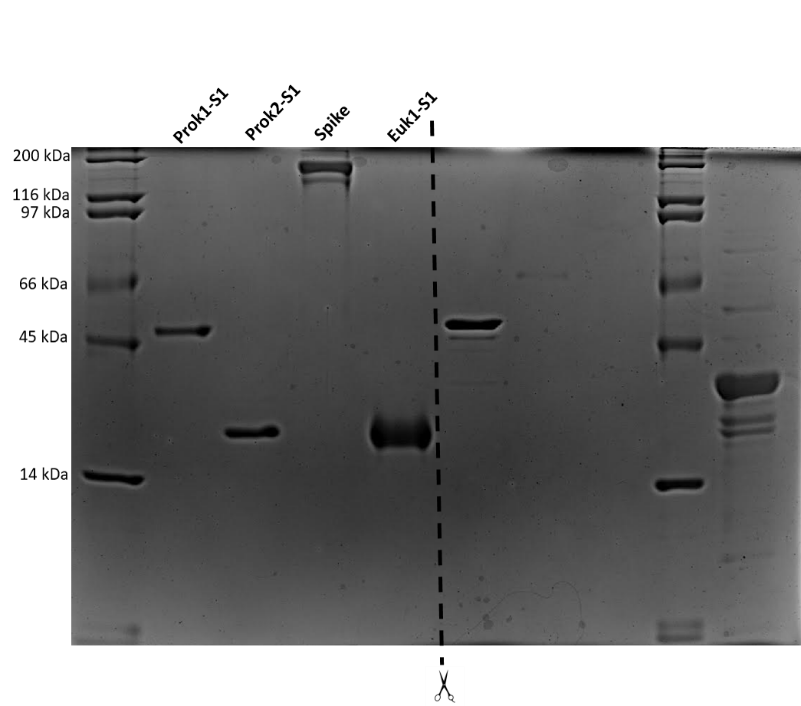


**Supplementary Figure 1:** Recombinant SARS-CoV-2 Spike proteins: Prok1-S1, Prok2-S1 and Euk1-S1. A) The total length of SARS-CoV-2 S is 1273 amino acids and consists of a signal peptide (amino acids 1–13), the S1 subunit (14–685 residues), and the S2 subunit (686– 1273 residues). The RBD (Receptor Binding Domain) encompassing amino acids 319-541 in the S1 domain. Amino acid sequence of the SARS-CoV-2 Spike protein: Prok1-S1 (250-667aa) in red > Prok2-S1 (319-591aa) underlined > Euk1-S1 (330-554aa) in bold. B) Full-length SDS-PAGE of the recombinant proteins.


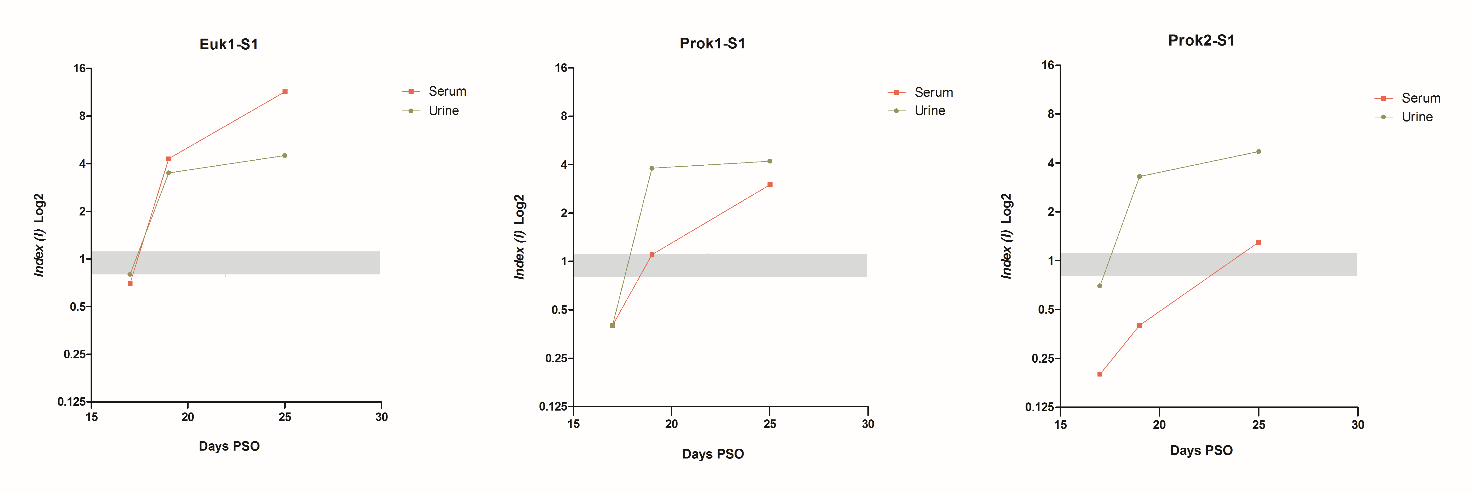


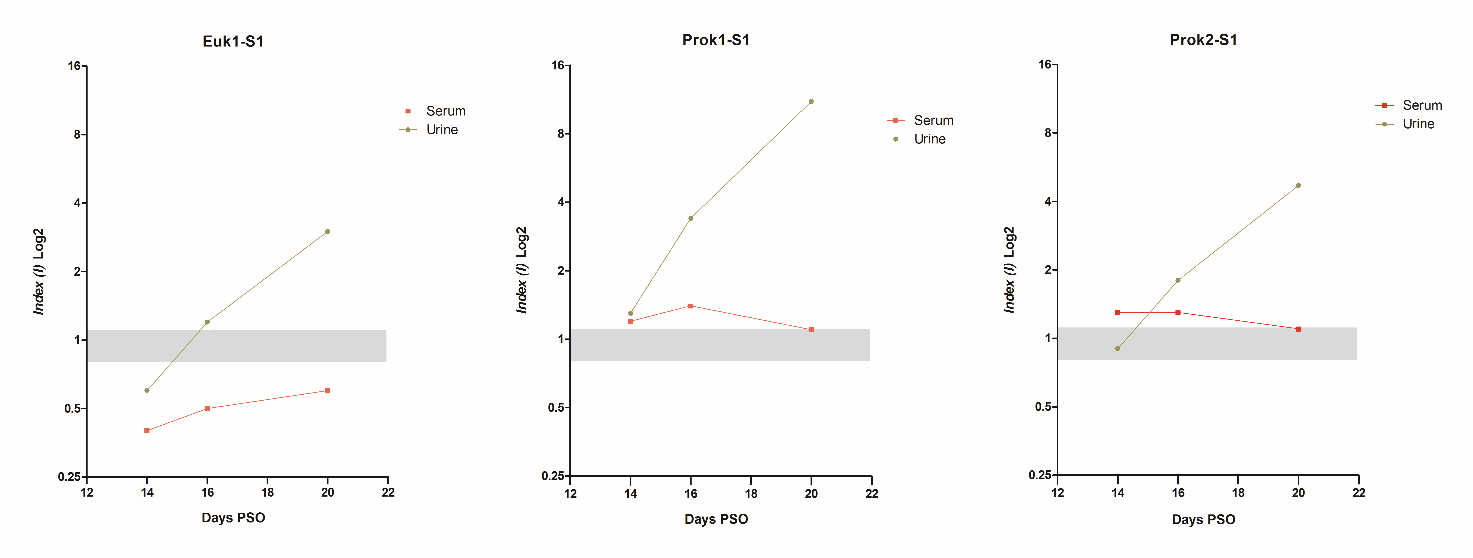


**Supplementary Figure 2. Dynamics of IgG antibody conversion in patient urine and serum samples.** Figures show the IgG levels specific to the recombinant SARS-CoV-2 Spike proteins (Euk1-S1, Prok1-S1, Prok2-S1) for two patients, with longitudinal collection on different days post-symptom onset. The plotted index values ​​(I) were related to the absorbance ratio on the cut-off.


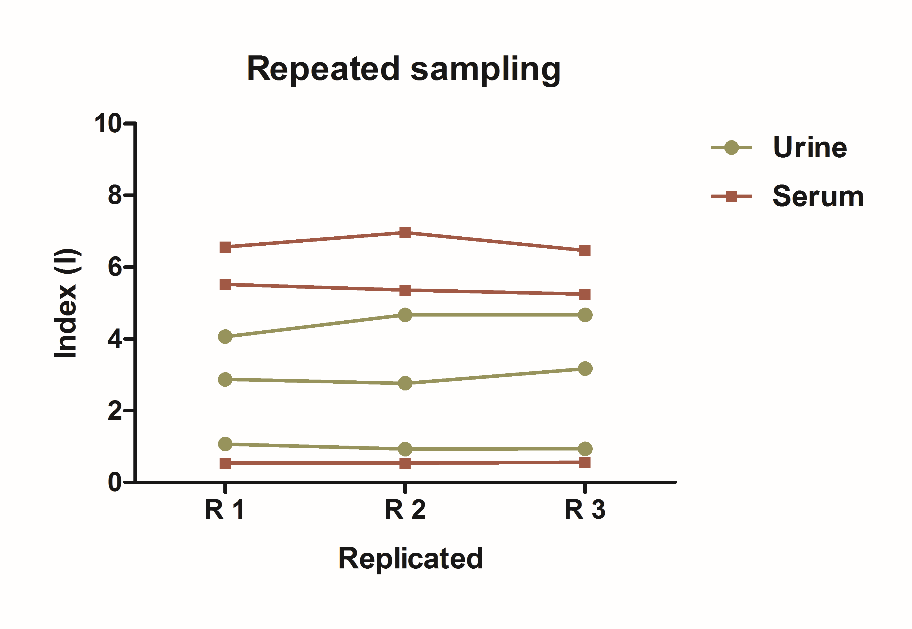


**Supplementary Figure 3. Experimental replication from same patient.** Experimental replication (R1, R2, R3) where conducted for three randomly individuals.


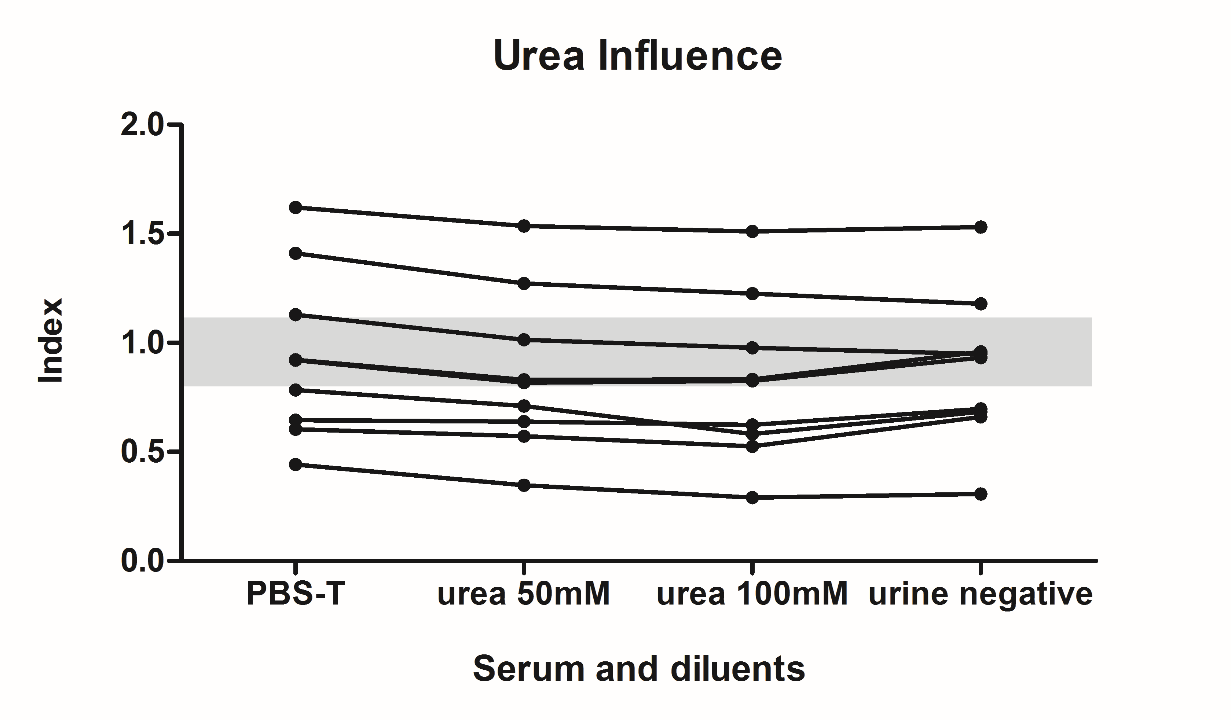


**Supplementary Figure 3. The influence of urea in serum based ELISA.** ELISA assay using the Prok2-S1 and serum samples diluted (1:100) in PBS-T, PBS-T 50mM Urea, PBS-T 100mM urea and also in urine sample collected before the pandemic.
